# Correlation analysis between the pulmonary function test and the radiological parameters of the main right thoracic curve in adolescent idiopathic scoliosis

**DOI:** 10.1186/s13018-019-1451-z

**Published:** 2019-12-16

**Authors:** Yonggang Wang, Fengguang Yang, Dongmin Wang, Haiyan Zhao, Zhanjun Ma, Peifen Ma, Xuchang Hu, Shixiong Wang, Xuewen Kang, Bingren Gao

**Affiliations:** 1 Department of Orthopedics, Orthopedics Key Laboratory of Gansu Province, Lanzhou University Second Hospital, No. 82 Cuiyingmen, Lanzhou, 730030 Gansu Province China; 2 Department of Cardiac Surgery, Lanzhou University Second Hospital, No. 82 Cuiyingmen, Lanzhou, 730030 Gansu Province China; 3 Lanzhou University, No. 222 South Tianshui Road, Lanzhou, 730000 Gansu Province China; 4 The International Cooperation Base of Gansu Province for the Pain Research in Spinal Disorders, No. 222 South Tianshui Road, Lanzhou, 730000 Gansu Province China; 5 Medical College of Northwest Minzu University, No. 1 Northwest xincun, Lanzhou, 730030 Gansu Province China; 6 Department of Orthopedics, The First Hospital of Lanzhou University, No.1 Donggang West Road, Lanzhou, 730000 Gansu Province China

**Keywords:** AIS, Adolescent idiopathic scoliosis, Pulmonary function

## Abstract

**Background:**

Scoliosis causes thoracic deformities, and it is necessary to assess these changes in pulmonary function test (PFT). To determine how measurements of spinal and thoracic cage deformities are related to pulmonary function.

**Methods:**

Seventy-two patients with main right thoracic curvature in adolescent idiopathic scoliosis (AIS) underwent a PFT and a radiological parameter evaluation of spinal and thoracic cage deformities. Simple and multiple linear regressions were also used to note whether a combination of variables might better predict PFT values. Means were compared using the two-sample *t* test or one-way ANOVA with Tukey’s multiple comparison methods.

**Results:**

Forced vital capacity (FVC)% predicted had significantly negative correlations with main thoracic curve Cobb (MT-Cobb) (*R*^2^ = 0.648, *p* < 0.001), main thoracic curve-rib hump (MT-RH) (*R*^2^ = 0.522, *p* < 0.001), main thoracic curve apical vertebral body-to-rib ratio (MT-AVB-R) (*R*^2^ = 0.536, *p* < 0.001), and main thoracic curve apical vertebra translation (MT-AVT) (*R*^2^ = 0.383, *p* < 0.001). Multiple regression analysis was performed with FVC% predicted as the dependent variable and MT-Cobb, MT-RH, MT-AVB-R, and MT-AVT as the independent variables. MT-Cobb, MT-RH, MT-AVB-R, and MT-AVT were factors with a significant effect on FVC% predicted (*p* < 0.001). For 45 patients who had preoperative FVC impairment (FVC% predicted < 80%), their MT-Cobb averaged 76.71°. Twenty-seven patients with normal preoperative FVC (FVC% predicted ≥ 80%) had a smaller mean MT-Cobb of 52.03° (*p* < 0.001). In other radiological parameters, the impaired FVC group had a MT-AVT of 54.29 mm compared to 38.06 mm for the normal FVC group (*p* < 0.001). MT-AVB-R averaged 2.92 for the impaired FVC group and 1.78 for the normal FVC group (*p* < 0.001). MT-RH averaged 28.79 mm for the impaired group and 16.62 mm for the normal group (*p* < 0.001). Further stratification of preoperative PFT results is divided into three groups. The three groups also showed significant differences in MT-Cobb, MT-RH, MT-AVB-R, and MT-AVT (*p* < 0.001).

**Conclusion:**

Severe scoliosis leads to an increased degree of thoracic deformity, which increases the risk of lung damage in AIS. Moreover, a more accurate assessment of pulmonary function is achieved through radiological parameters and PFTs.

## Background

Scoliosis is a common musculoskeletal disease of the spine. It is a generic term for a group of different diseases of the spine, thoracic and trunk shape and position caused by a change in composition [1–3]. It is characterized by observable lateral bending, which has a direct impact on the thoracic cavity [4, 5]. The Scoliosis Research Society (SRS) recommends that the diagnosis is confirmed when the Cobb’s angle is 10° or higher and axial rotation can be identified [6]. However, when the reasons of scoliosis are unknown, these patients are diagnosed with idiopathic scoliosis (IS) [7].

Scoliosis has a large impact on the patient. In addition to back pain, health-related quality of life, and psychosocial and cosmetic problems, severe thoracic curves are also obviously associated with decreased cardiopulmonary function and increased mortality [1, 8, 9]. In general, the progression of thoracic scoliosis can lead to the deterioration of pulmonary function [10, 11]. The spinal deformity and its effect on pulmonary function have gained widespread attention. However, there is less focus on the assessment of lung function, which could also be impaired due to the biomechanical changes of the spine and thoracic cage [12, 13]. Therefore, in patients with scoliosis, especially those with chest curvature, predicting and interfering with pulmonary function are particularly important. In previous studies, the correlation between scoliosis and pulmonary function was assessed mainly on the scoliosis features of the coronal, sagittal, and axial planes and even three-dimensional analysis [2, 14–20]. These scoliosis features are expressed by radiological parameters, which exist independently and rarely reflect the common changes of the spine and thorax.

This study was designed to assess the relationship between adolescent idiopathic scoliosis (AIS) and pulmonary function. We inferred the characteristics of scoliosis based on the impairment of different pulmonary functions. We investigated which radiological parameter is the more accurate predictor of lung damage. In addition to main thoracic curve apical vertebra translation (MT-AVT), main thoracic curve apical vertebral body-to-rib ratio (MT-AVB-R) was determined in the study because in AIS, thoracic deformity is caused by deformity of the spine. Severe thoracic deformity eventually leads to decreased pulmonary function. The MT-AVB-R is a previously undescribed measurement that is related to pulmonary function. However, in addition to assess thoracic apical vertebrae translation, the MT-AVB-R provides assessment of the overall thoracic and rib deformity.

## Materials and methods

From September 2015 to August 2019, the data of 60 AIS patients who needed surgical treatment were collected in the Spinal Surgery Department of the Lanzhou University Second Hospital. From January 2017 to August 2019, the data of 12 AIS patients who needed surgical treatment were collected at the Spinal Surgery Department of Gansu Provincial People’s Hospital. The total sample size was 72 cases. Preoperative data of patients included demographic data (age and sex, BMI), pulmonary function tests (PFTs), standing anteroposterior, standing lateral, and supine bending radiographs of the whole spine. Inclusion criteria are as follows: (1) AIS patients; (2) main thoracic curve ≥ 45°; (3) right thoracic scoliosis; (4) no other lung diseases; and (5) Lenke types 1, 2, 3, 4, and 6. We excluded patients with obesity because obesity causes deleterious effects on the lung volume and capacity in children and adolescents [21, 22]. In addition to obese patients, we also excluded patients with respiratory failure and patients with primary lung diseases affecting lung function, such as asthma and bronchitis.

All radiographic data were measured from PACS by two senior spine surgeons. The following are the specific measurement methods and evaluation parameters [23–25].

- Main thoracic curve Cobb’s angle (MT-Cobb): In a standing posterior-anterior (PA) radiograph, the angle is composed of two intersecting lines: the upper endplate of the main thoracic curve upper vertebra and the lower endplate of the main thoracic curve lower vertebra [24, 25].
- Main thoracic curve-flexibility index (MT-FI): On the preoperative supine bending radiographs, the main thoracic curves were measured. The main thoracic flexible index (MT-FI) was then calculated [25]:
  $$ \mathrm{FI}\left(\%\right)=\frac{\mathrm{Standing}\ \mathrm{coronal}\ \mathrm{Cobb}^{\prime}\mathrm{s}\ \mathrm{angle}-\mathrm{Supine}\ \mathrm{bending}\ \mathrm{Cobb}^{\prime}\mathrm{s}\ \mathrm{angle}}{\mathrm{Standing}\ \mathrm{coronal}\ \mathrm{Cobb}^{\prime}\mathrm{s}\ \mathrm{angle}}\times 100\% $$
- Main thoracic curve-apical vertebral body-to-rib ratio (MT-AVB-R): The ratio of linear measurements from the lateral borders of the main thoracic apical vertebrae to the chest wall on anterior-posterior radiographs [26, 27].
- Main thoracic curve -apical vertebra translation (MT-AVT): The horizontal distance from the midpoint of the main thoracic curve apical vertebra (or intervertebral disc) to the C7PL [25, 27].
- Main thoracic curve-rib hump (MT-RH): The linear distance between the left and right posterior rib prominences at the apex of the main thoracic curve apical vertebra rib deformity on a lateral radiograph [26].
- Thoracic kyphosis (TK): In the sagittal plane, thoracic kyphosis from T5 to T12. In a standing X-ray lateral image, the angle is composed of two perpendicular lines: the upper endplate of the T5 vertebra and the lower endplate of T12 [23, 25].
- Main thoracic curve-thoracic depth (MT-TD): The linear distance between the anterior edge of the vertebral body and the sternum in the apical vertebra region of the main thoracic curve [25].All patients completed standard PFT before surgery. Severe scoliosis leads to limited expansion of the chest and lungs and restrictive ventilation disorders, mainly manifested as a significant decrease in forced vital capacity (FVC)% predicted. Pulmonary function was considered to be impaired when FVC% predicted was < 80% of the predicted values [28, 29]. In the preoperative PFT results, we mainly choose FVC% predicted value and forced expiratory volume in 1 s (FEV_1_)% predicted value. Plethysmography and pulmonary function testing were used to measure FVC and FEV_1_. Each test was repeated three times, and the single best effort was recorded. The following parameters were evaluated:
- Forced vital capacity (FVC): Maximum volume of air expelled as rapidly and completely as possible by a maximum effort after a maximum inspiration.
- Forced expiratory volume in 1 s (FEV_1_): The amount of air expired in 1 s during a rapid and maximal expiration from full inspiration.

## Statistical methods

Preoperative PTF and radiological parameters were measured in all 46 patients. Descriptive statistics reported the mean and standard deviation. We conducted a correlation analysis and determined a Pearson correlation coefficient (*r*) between the pulmonary function parameters and all of the radiographic measurements of the spinal deformity. The percentages of the predicted values for each of the pulmonary function parameters were used instead of the absolute values because these values were controlled for age, height, and gender, thereby eliminating those factors as possible confounding variables in the analysis. Radiographic factors that had significant *r* values (*p* < 0.01) were entered into a stepwise multiple regression analysis, and the coefficient of multiple determination (*R*^2^) was calculated.

Because scoliosis can lead to restrictive pulmonary dysfunction, interpretative strategies for PFTs at ATS/ERS 2005 were used to separate the entire cohort into two groups [28, 29]. According to severity of any spirometry abnormality based on the FEV_1_% [28, 29], preoperative PFT was further stratified into three groups. We then correlated the preoperative radiographic measurements—MT-Cobb, MT-FI, MT-AVB-R ratio, MT-AVT, MT-RH, TK (T5 to T12), and MT-TD—with the PFT results. Means were compared using the two-sample *t* test (assuming unequal variances) or one-way ANOVA with Tukey’s multiple comparison methods if three or more means were being compared. A *p* < 0.05 was considered statistically significant. SPSS statistical software (IBM SPSS Statistics version 25) was used.

## Results

Gender, age, standing height and weight, and BMI are listed in Table 1. In all AIS patients, the radiological characteristics were the right main thoracic curve. The most common curve pattern was Lenke type 1, which was found in 41 patients (56.9%). Lenke type 2 and Lenke type 3 were the second and third most common curve patterns, occurring in 14 (19.4%) and 12 (16.7%) of the patients, respectively. The remaining 5 patients had a Lenke type 6 (6.9%). The apical vertebrae of the main thoracic curve had 11 cases of T7, 17 cases of T8, 31 cases of T9, 6 cases of T10, and 7 cases of T11. The coronal plane deformities of the thoracic spine and the sagittal plane measurements are shown in Table 2.

**Table 1:** Human characteristics data of patients with AIS

	Male, *N* = 17 (23.6%)		Female, *N* = 55 (76.4%)	
	Mean ± SD	Range	Mean ± SD	Range
Age (year)	14.71 ± 0.59	10–18	14.85 ± 0.36	10–18
Height (cm)	155.12 ± 3.39	126–171	151.56 ± 1.51	105–170
Weight (kg)	46.12 ± 1.97	31–58	43.00 ± 1.07	18–66
Body mass index (kg/m^2^)	19.35 ± 0.65	15–24	18.65 ± 0.34	13–23

**Table 2:** Radiological and PFT data of patients with AIS

Radiological and PFT	Mean ± SD
MT-Cobb (°)	67.46 ± 17.62
TK(T5-T12)(°)	29.27 ± 18.91
MT-RH (mm)	24.23 ± 8.90
MT-AVT (mm)	48.21 ± 16.90
MT-AVB-R	2.49 ± 1.11
MT-TH (mm)	82.52 ± 23.69
MT-FI	0.37 ± 0.23
FVC% predicted	74.41 ± 20.25%
FEV_1_% predicted	75.32 ± 19.80%

The mean and standard deviation of the radiological and PFT results are shown in Table 2. Restrictive ventilation disorder with FVC% predicted < 80% was 45 patients (62.5%). According to interpretative strategies for PFT at ATS/ERS 2005, the degree of damage was 34 patients with no pulmonary impairment, 22 patients with mild and moderate impairment, and 16 patients with severe impairment.

**Fig. 1:**
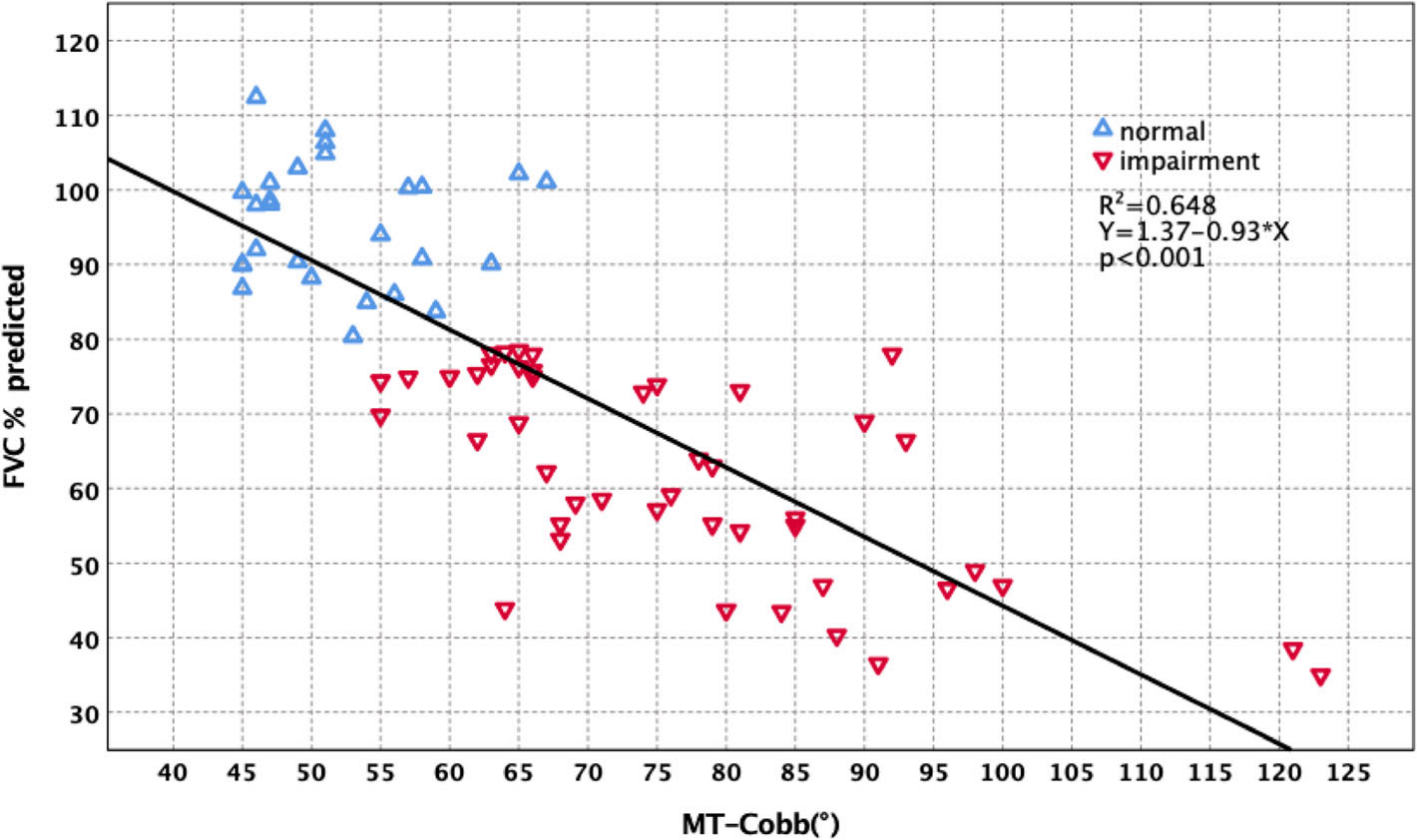
Effect and correlation of MT-Cobb on pulmonary function impairment

**Fig. 2:**
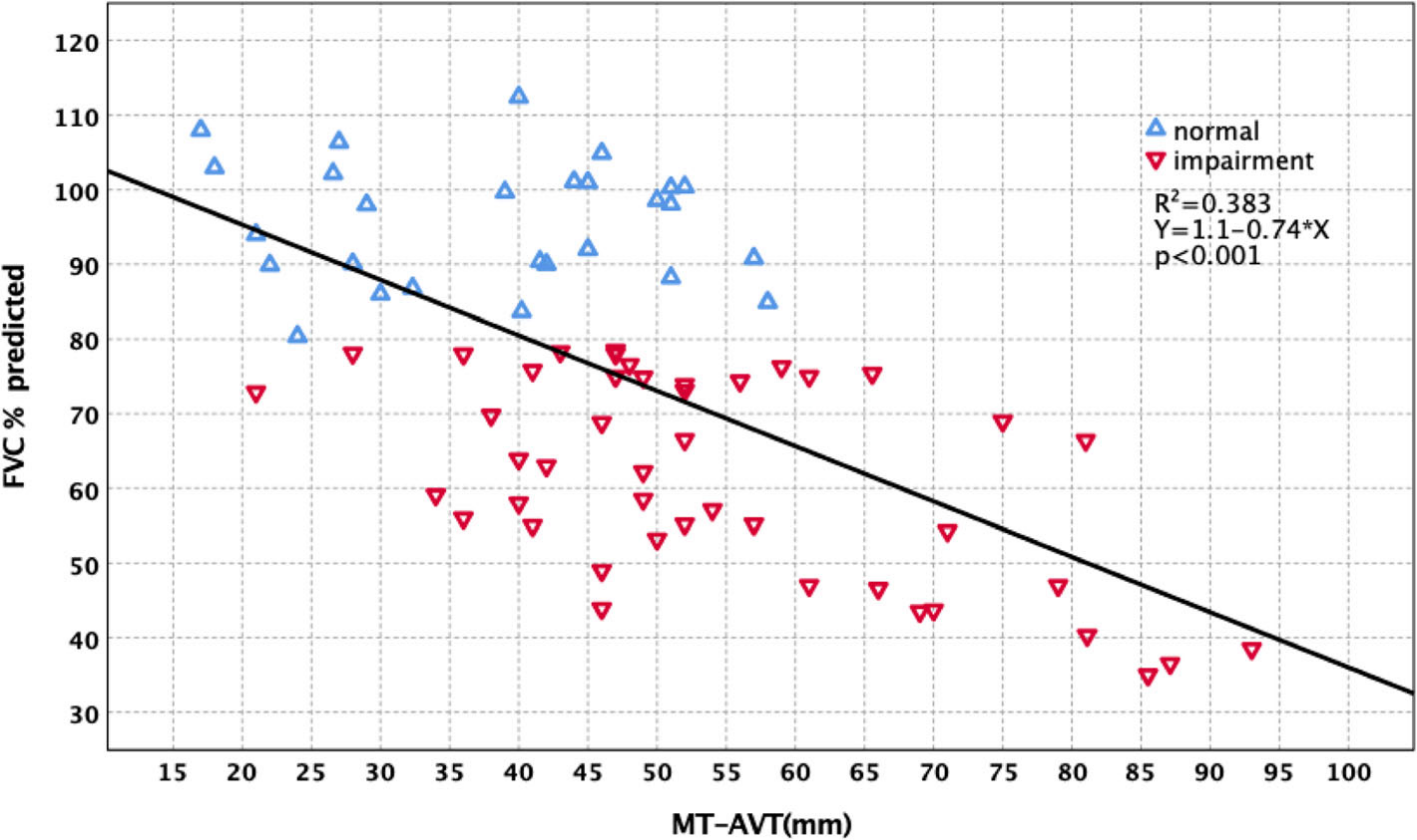
Effect and correlation of MT-AVT on pulmonary function impairment

**Fig. 3:**
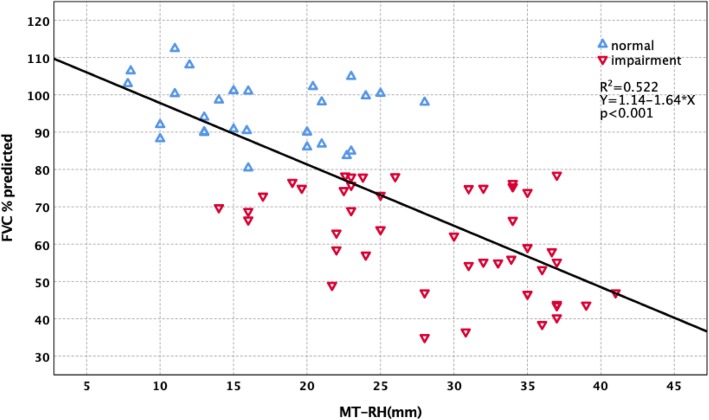
Effect and correlation of MT-RH on pulmonary function impairment

**Fig. 4:**
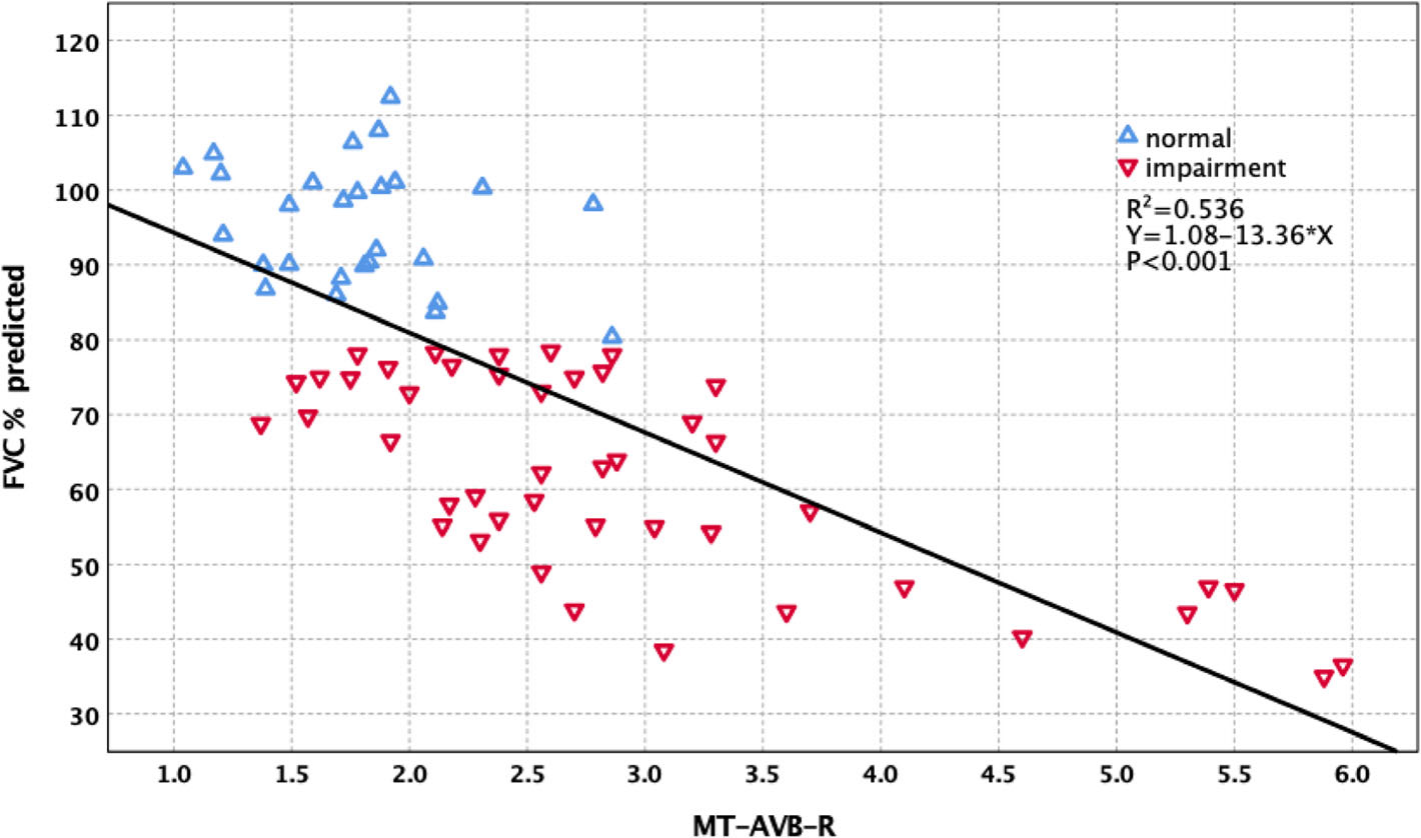
Effect and correlation of MT-AVB-R on pulmonary function impairment

The correlation between PFT and radiological parameters are shown in Table 3. FVC% predicted had significantly negative correlations with MT-Cobb (*R*^2^ = 0.648, *p* < 0.001) (Fig. 1), MT-AVT (*R*^2^ = 0.383, *p* < 0.001) (Fig. 2), MT-RH (*R*^2^ = 0.522, *p* < 0.001) (Fig. 3), and MT-AVB-R (*R*^2^ = 0.536, *p* < 0.001) (Fig. 4). MT-FI, TK (T5 to T12) and MT-TD showed little correlation and no difference with FVC% predicted. Multiple regression analysis using the stepwise method was performed with FVC% predicted as the dependent variable and MT-Cobb, MT-TD, MT-RH, and MT-AVB-R as the independent variables. MT-Cobb, MT-TD, MT-RH, and MT-AVB-R had a significant effect on FVC% predicted (*p* < 0.05). FVC% predicted can be predicted from the MT-Cobb, MT-TD, MT-RH, and MT-AVB-R.

**Table 3:** The correlation between PFT and radiological parameters

Radiological parameters	Correlation			
	FEV1% predicted (*r*)	*p*	FVC% predicted (*r*)	*p*
MT-Cobb	− 0.812***	< 0.001	− 0.805***	< 0.001
MT-AVB-R	− 0.748***	< 0.001	− 0.732***	< 0.001
MT-AVT	− 0.670***	< 0.001	− 0.619***	< 0.001
MT-RH	− 0.756***	< 0.001	− 0.723***	< 0.001
TK(T5–T12)	− 0.248**	0.036	− 0.172	0.147
MT-TD	0.227	0.055	0.269**	0.022
MT-FI	0.233**	0.048	0.207	0.81

**p < 0.05

***p < 0.001

For 45 patients who had preoperative FVC impairment (< 80% predicted), their MT-Cobb curves averaged 76.71°. Twenty-seven patients with normal preoperative FVC (≥ 80% predicted) had a significantly smaller mean MT-Cobb of 52.03° (*p* < 0.001). In other radiological parameters, the impaired FVC group had a MT-AVT of 52.29 mm compared to 38.06 mm for the normal FVC group (*p* < 0.001). MT-AVB-R averaged 2.92 for the impaired group and 1.78 for the normal group (*p* < 0.001). MT-RH averaged 28.79 mm for the impaired group and 16.62 mm for the normal group (*p* < 0.001). Thus, patients with preoperative FVC impairment had significantly larger MT-Cobb, MT-AVT, MT-AVB-R, and MT-RH than those with normal FVC (Table 4).

**Table 4:** Radiographic parameters of scoliosis and FVC% predicted

Radiographic parameters	FVC% predicted	*N*	Mean ± SD	Min	Max	*p*
MT-Cobb	FVC% predicted ≥ 80%	27	52.03 ± 1.25	45.00	67.00	< 0.001
	FVC% predicted < 80%	45	76.71 ± 2.32	55.00	123.0	
MT-AVT	FVC% predicted ≥ 80%	27	38.06 ± 2.37	17.00	58.00	< 0.001
	FVC% predicted < 80%	45	54.29 ± 2.44	21.00	93.00	
MT-AVB-R	FVC% predicted ≥ 80%	27	1.78 ± 0.0.84	1.04	2.86	< 0.001
	FVC% predicted < 80%	45	2.92 ± 0.17	1.37	5.96	
MT-RH	FVC% predicted ≥ 80%	27	16.62 ± 1.08	7.80	28.00	< 0.001
	FVC% predicted < 80%	45	28.79 ± 1.08	14.00	41.00	

Preoperative PFT further stratified the patients into three groups: no pulmonary impairment (FVC% predicted ≥ 80%); for FVC% predicted < 80%, patients were divided into mild or moderate impairment (FEV_1_% predicted ≥ 60%) and severe impairment (FEV_1_% predicted < 60%). Three groups also showed significant differences in MT-Cobb, MT-AVT, MT-AVB-R, and MT-RH (*p* < 0.001, Table 5).

**Table 5:** Radiographic parameters of scoliosis and FEV_1_% predicted

Radiographic parameters	PTF groups	*N*	Mean ± SD	Min	Max	*p*
MT-Cobb’s angles	No pulmonary impairment	34	56.06 ± 1.90	45.00	92.00	< 0.001
	Mild or moderate impairment	22	69.59 ± 2.21	55.00	93.00	
	Severe impairment	16	88.75 ± 4.07	64.00	123.00	
MT-AVT	No pulmonary impairment	34	38.86 ± 2.11	17.00	58.00	< 0.001
	Mild or moderate impairment	22	52.26 ± 2.43	36.00	81.00	
	Severe impairment	16	63.86 ± 4.76	34.00	93.00	
MT-AVB-R	No pulmonary impairment	34	1.85 ± 0.84	1.04	2.86	< 0.001
	Mild or moderate impairment	22	2.46 ± 0.12	1.57	3.70	
	Severe impairment	16	3.89 ± 0.34	2.28	5.96	
MT-RH	No pulmonary impairment	34	17.71 ± 0.98	7.80	28.00	< 0.001
	Mild or moderate impairment	22	27.95 ± 1.59	14.00	37.00	
	Severe impairment	16	32.96 ± 1.32	21.00	41.00	

## Discussion

Many studies have indicated the reduction in pulmonary function caused by spinal deformities. A spinal deformity is a change in the coronal and sagittal planes [17], as well as the three-dimensional structure [19]. Specifically, this change is related to the decline in pulmonary function with the increase in the severity of spinal deformity [14–16, 18, 30–34]. In the above studies, the effects of radiological parameters of spinal deformities and pulmonary function have been extensively studied, such as MT-Cobb, MT-AVT, TK, and MT-RH. However, there are few studies on the effects of other radiological parameters of spinal deformity and pulmonary function, for example, MT-AVB-R, MT-FI, and MT-TD.

Previous literature has shown that MT-Cobb, MT-AVT, and MT-RH have a significantly negative correlation with lung function. However, for TK, MT-FI also had significant but weak correlations with pulmonary function [17, 18, 31, 35–37]. Similarly, our research also confirmed this relationship.

We tried to identify associations between radiological parameters (MT-Cobb, MT-AVB-R, MT-TD, T-AVT, TK, MT-RH, and MT-FI) and pulmonary function in AIS. Our primary finding was that MT-Cobb, MT-AVB-R, MT-AVT, and MT-RH were negatively correlated with lung function, which was statistically significant (*p* < 0.001) (Table 3). The relationship between MT-AVB-R and pulmonary function has not been reported in previous studies. We found that MT-AVB-R has the obvious correlation with pulmonary function and is closed to the correlation between pulmonary function and MT-Cobb. If the lung function was predicted by MT-AVB-R, it has the characteristics of simple measurement and small error [26, 27]. The data suggested that MT-Cobb, MT-AVB-R, MT-AVT, and MT-RH contribute to pulmonary impairment in AIS patients.

MT-FI and TK were less correlated than their individual relationships with PFT and were not statistically significant. Additionally, they contributed independently to PTF, with linear regression suggesting that the relationship of flexibility and TK to PTF may not be entirely mediated through Cobb. Upadhyay et al. showed that T-FI and TK were not correlated with PTF [35]. However, a large multi-center database of surgically treated AIS patients with Lenke 1 to Lenke 4 curves was queried to report preoperative PFT and correlation with severity of MT curve and sagittal plane hypokyphosis (T5–T12 < 10°) [18]. In our research, there was a low correlation between TK and vital capacity. On the other hand, it is difficult to display the upper thoracic region on chest radiographs or old spine radiographs [23], which is prone to errors in measurement.

The correlation between thoracic spinal deformity and decreased pulmonary function in AIS has been suggested in previous research. Newton et al. found the magnitude of the thoracic curve, numerous vertebrae involved in the thoracic curve, thoracic hypokyphosis, and coronal imbalance to be associated with an increased risk of moderate or severe pulmonary impairment [17]. Similarly, our study found MT-Cobb, MT-AVB-R, MT-ATV, and MT-RH to be statistically significant as predictors of FVC% predicted. The data suggested that MT-Cobb, MT-AVT, MT-AVB-R, and MT-RH contributed to pulmonary impairment in AIS patients.

We classified the patients according to the change in FVC% into the no impairment group (FVC% > 80%) and impairment group (FVC% < 80%). The radiological parameters (MT-Cobb, MT-RH, MT-AVT, and MT-AVB-R) associated with lung function impairment were compared and found to be statistically significant. We can conclude that when spinal deformity develops to a certain extent, lung function will be impaired (Table 4). According to ATS/ERS 2005, pulmonary function impairment was classified into three groups, the correlative radiological parameters were compared, and the results were statistically significant. Therefore, we should attempt to classify the severity of scoliosis according to the severity of pulmonary impairment (Table 5).

Our study found that scoliosis severity responds to the severity of pulmonary function. Through this rule, changes in pulmonary function can be well assessed by changes in pulmonary function via using relevant radiological parameters.

One of the limitations of this study is obtaining from the reliability of PFT [28, 29], because the test for lung function is affected by the quality requirements of the operator, subjective and objective factors of the subject, and environmental factors. The results of the PTFs were not corrected for the reduction in height due to scoliosis. In addition, the sample size in this study was small and had a statistical impact. In the future, we intend to cooperate with other hospitals to conduct multi-center clinical research and expand the sample size, and further to validate our research.

## Conclusion

Severe scoliosis leads to an increased degree of thoracic deformity, which increases the risk of lung damage in AIS. In orthopedic surgery, surgeons should pay attention to improve in the appearance of patients with scoliosis, as well as in lung function and the effect of surgery on lung function. This requires the surgeon to evaluate MT-Cobb, MT-RH, MT-AVB-R, and MT-AVT, not only to increase the improvement of scoliosis but also to increase the improvement of thoracic deformity. Moreover, a more accurate assessment of pulmonary function is achieved through radiological parameters and PFT.

## Data Availability

All data were true and effective, and all patients were hospitalized in our hospital.

## Abbreviations

AIS: Adolescent idiopathic scoliosis
BMI: Body mass index
C7PL: The seventh cervical vertebra plumb line
FEV1: Forced expiratory volume in 1 s
FVC: Forced vital capacity
MT-AVB-R ratio: Main thoracic curve apical vertebral body-to-rib ratio
MT-AVT: Main thoracic curve apical vertebra translation
MT-Cobb: Main thoracic curve Cobb’s angle
MT-FI: Main thoracic curve-flexibility index
MT-RH: Main thoracic curve-rib hump
MT-TD: Main thoracic curvet thoracic depth
PACS: Picture archives and communications system
PFT: Pulmonary function test
TK: Thoracic kyphosis
